# Probiotic Supplementation Can Alter Inflammation Parameters and Self-Reported Sleep After a Marathon: A Randomized, Double-Blind, Placebo-Controlled Study

**DOI:** 10.3390/nu17233762

**Published:** 2025-11-29

**Authors:** Valdir Aquino-Lemos, Geovana S. F. Leite, Edgar T. Silva, Helena A. P. Batatinha, Ayane S. Resende, Antônio H. Lancha-Junior, José C. R. Neto, Sergio Tufik, Ronaldo V. Thomatieli-Santos

**Affiliations:** 1 Department of Bioscience, Federal University of São Paulo, Santos 11015-020, SP, Brazil; 2 School of Physical Education, University of São Paulo, São Paulo 05508-030, SP, Brazil; 3 Department of Psychobiology, Federal University of São Paulo, São Paulo 04040-003, SP, Brazil

**Keywords:** strenuous physical exercise, sleep, sleepiness, marathon, probiotics, gut–brain axis

## Abstract

**Background:** Sleep is essential for athletes’ physical performance and recovery. However, strenuous exercise has the potential to increase inflammation and worsen sleep. This study evaluated the effect of probiotic supplementation on self-reported sleep quality, daytime sleepiness, and inflammatory profile 24 h after a marathon. **Methods**: 27 marathon male runners were divided into the Probiotic group (Probiotic n = 14) or the Placebo group (Placebo n = 13). The Probiotic group consumed 1 × 10^10^ CFU of Lactobacillus acidophilus, 1 × 10^10^ CFU of Bifidobacterium lactis + 5 g/day maltodextrin for 30 days prior to the marathon. The Placebo group received a sachet of 5 g/day maltodextrin during the same period. Sleep and inflammatory status were assessed before supplementation, before the marathon, and 1 h and 24 h after the marathon. Data were analyzed using Statistic 13.3 and expressed as mean ± standard deviation. Tukey’s post hoc test was followed by a two-way ANOVA with repeated measures. The level of significance was set at *p* ≤ 0.05. **Results**: In the Placebo group, daytime sleepiness, sleep latency, and global sleep score increased 24 h after the marathon, while total sleep time and sleep efficiency decreased. In the Probiotic group, daytime sleepiness, sleep latency, and global sleep scores were lower 24 h after the marathon compared to the Placebo group. Total sleep time and sleep efficiency were higher in the Probiotic group compared to the Placebo group 24 h after the marathon. IL-1β and TNF-α concentrations decreased compared to Basal in both groups. IL-1β levels were lower 24 h after treatment compared to pre-treatment in the Placebo group. IL-6 was lower 24 h after the marathon in both groups. LPS concentrations were lower 1 h and 24 h after the marathon in the Probiotic group compared to the Basal group. There was no difference in cytokines and LPS between the groups. **Conclusions**: Supplementation with *Lactobacillus acidophilus* and *Bifidobacterium lactis* for 30 days changes self-reported sleep and reduces LPS concentration after the marathon.

## 1. Introduction

Sleep is essential for maintaining physiological and cognitive functions, and it is crucial for physical performance and recovery in athletes [1,2]. Conversely, strenuous physical exercise, such as marathon running, increases sleep latency, reduces total sleep time and efficiency, and increases daytime sleepiness [3,4]. Sleep quality is often impaired following marathon and ultra-endurance races, characterized by reduced sleep efficiency and increased nighttime awakenings during the first night after races [3]. A recent study suggests that a marathon is likely to induce more sleep disturbances than a training session or no exercise at all. However, this field considers some findings to be controversial [4].

Recently, it has been discussed that gut microbiota may also influence sleep through peripheral mechanisms. Indeed, communication between the gut and the brain involves neural, immunological, and metabolic pathways that can influence circadian rhythm and sleep quality [5,6]. Intestinal dysbiosis influences intestinal permeability, including the release of lipopolysaccharides (LPSs), which contributes to systemic inflammation, neuroinflammation, and impaired sleep [5,7,8].

During prolonged exercise, blood flow redistribution and heat stress compromise intestinal barrier integrity, leading to increased serum LPS concentrations [9,10]. The activation of inflammatory and neuroendocrine pathways due to increased LPS may also partially explain the sleep alterations promoted by strenuous exercise [11,12,13]. Recent studies have suggested that probiotic supplementation may be a promising strategy for restoring intestinal homeostasis and modulating systemic inflammatory responses [14]. Supplementation with *Lactobacillus acidophilus* and *Bifidobacterium lactis* can help preserve the intestinal barrier and reduce bacterial translocation [15,16]. Thus, it changes the pro- and anti-inflammatory balance, reducing inflammatory pathways and enhancing sleep [17,18,19].

Despite the growing interest in the role of microbiota in sleep regulation, there is still no direct evidence, and studies have not been conducted in the context of physical exercise. Consequently, there is still insufficient robust evidence on the effectiveness of probiotic supplementation in mitigating sleep impairment associated with strenuous physical exercise, especially long-duration exercise such as marathons. This study proposes that 30-day probiotic supplementation may mitigate the effects of marathons on sleep and inflammation. This study evaluated the effect of probiotic supplementation on sleep quality and daytime sleepiness self-reported in runners 24 h after a marathon. We hypothesized that probiotics may preserve the integrity of the intestinal barrier, reduce systemic inflammation, and, through communication between the gut and the brain, mitigate sleep impairment self-reported induced by marathons.

## 2. Methods

**Participants:** This study was approved on 27 June 2017 by the UNIFESP/Hospital São Paulo Research Ethics Committee under No. 2,880,287. The runners had experience running marathons and trained at least five times per week. Volunteers with psychological disorders or undergoing psychiatric treatment, smokers, alcohol or illicit drug users, and health problems (cardiovascular, respiratory, chronic degenerative diseases, psychological disorders, or psychiatric treatment as assessed by a physician) were excluded from the study. Furthermore, participants were excluded if they were taking any medications that could interfere with the study results. Participants were randomized into two different groups using the website www.randomizer.org: Probiotic (n = 14) or Placebo (n = 13). The sample size calculation drew inspiration from the study of IL-6 [20]. The sample size calculation for the repeated measures ANOVA (2 groups × 4 time points) was performed using G*Power software version 3.1.9.7. The significance level adopted was 0.05, and the statistical power was set at a value above 0.80. The estimated effect size was 0.47 for intra-individual factors and 0.62 for inter-group factors, with a repeated measures correlation of 0.25. Based on these parameters, the program indicated that a sample of ten participants per group would be sufficient to detect differences between the pre- and post-supplementation time points. Considering the particularities of the experimental design and possible individual variations, it was decided to start the study with a larger number of participants, resulting in thirteen volunteers in the Placebo group and fourteen in the Probiotic group.

**Figure 1:**
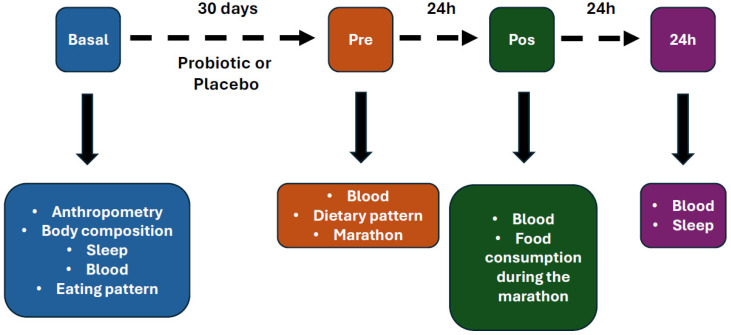
Experimental design.

**Design and Procedure:** Volunteers were assessed at four time points: (1) basal, (2) after thirty days of supplementation and 24 h before the marathon, (3) 60 min after the marathon, and (4) 24 h after the marathon. For this specific study, sleep quality and daytime sleepiness questionnaires were completed at timepoints 1 and 4. Nutritional assessment and body composition were completed at time points 1 and 2. Blood was collected at all timepoints (Figure 1).

**Sample Characterization:** Body mass was measured on a scale with a precision of 0.1 g. Height was measured using a vertical stadiometer with an accuracy of 1 mm. Body composition was determined using plethysmography with a BOD POD body composition system (Life Measurement Instruments, Concord, CA, USA).

**Supplementation:** Participants in the Probiotic-supplemented group received sachets containing 1 × 10^10^ CFU of *Lactobacillus acidophilus*, 1 × 10^10^ CFU of *Bifidobacterium lactis*, and 5 g/day of maltodextrin. The Placebo group received sachets containing 5 g/day of maltodextrin in colorless, tasteless powder form, with the same shape, color, odor, and size as the Probiotic-supplemented group. Both groups received supplementation for 30 days. The distribution of runners into the two groups was randomized, and all procedures were conducted in a double-blind manner for both the marathon runners and the researcher. During the supplementation period, participants were contacted by the principal investigator every two days to monitor for possible symptoms/adverse effects and to verify daily use of the supplementation. After the end of the supplementation period, the study’s confidentiality was tested by asking the volunteers if they knew what they were taking. All volunteers were instructed to ingest one sachet twice a day (on an empty stomach upon waking in the morning and in the evening, just before bed) to ensure the effectiveness of the supplementation used due to better adhesion of bacteria in transit. Participants were instructed to dilute the powder in 50 mL of water and shake until completely dissolved immediately before consumption. Physical Exercise Protocol: All participants completed an official marathon (42,195 m) in the shortest time possible, starting at 8:00 am. In the 30 days prior to the marathon, the volunteers trained approximately 50 km per week.

**Epworth Sleepiness Scale (ESS):** The ESS is a self-administered questionnaire that assesses the self-reported likelihood of dozing in eight everyday situations. To rate the likelihood of dozing, individuals use a scale from 0 (zero) to 3 (three), where zero corresponds to no likelihood of dozing and 3 to a high likelihood of dozing. Using a total score greater than 10 as the cutoff point, individuals with a high likelihood of excessive daytime sleepiness can be identified. Scores greater than 16 (sixteen) indicate severe sleepiness, most commonly found in patients with moderate or severe obstructive sleep apnea, narcolepsy, or idiopathic hypersomnia. The average score for a population without sleep disorders is 4 ± 3 points [21].

**Pittsburgh Sleep Quality Index (PSQI):** The self-reported questionnaire consists of 19 self-administered questions and five roommate-administered questions. The latter are used for clinical information only. The 19 questions are grouped into seven components, weighted on a Likert scale from 0 to 3. The PSQI components include subjective sleep quality, latency, duration, sleep efficiency, sleep disturbances, use of sleeping medications, and daytime dysfunction. An overall PSQI score of 0 to 4 is good, 5 to 10 is poor, and >10 indicates the presence of sleep disorders [22].

**Blood Collection**: 18 mL of blood was collected and distributed into tubes without an anticoagulant. Blood was collected at baseline, pre-marathon, and 24 h after the marathon (following an overnight fast, collected between 7:00 and 8:00 am), as well as 30 min after the participant finished the marathon. After collection, the blood was centrifuged at 400× *g* for 15 min at 4 °C. The serum was placed in plastic Eppendorf microtubes and stored in a freezer at −80 °C for later analysis.

**Serum Cytokines**: The concentrations of IL-1β, IL-6, and TNF-α were assessed using a multiplex assay with Millipore Darmstadt kits (Merck KGaA, Darmstadt, Germany) from Germany. The Luminex 200 Analyzer was used in conjunction with the Magpix system, utilizing Milliplex Analyst 5.1 software. All manufacturers’ specifications were followed. Intestinal permeability: Serum endotoxin (LPS) concentration was determined using an ELISA kit from Mybiosource, San Diego, CA, USA. All manufacturers’ specifications were followed.

**Self-Reported Dietary Pattern**: The participant completed food diaries on sheets of paper in the presence of an experienced researcher, who provided clarification upon request. To assess energy and macronutrient intake (carbohydrates, protein, and lipids), the ELSA-BRAZIL food frequency questionnaire [23] and a three-day food diary were administered. The Food Frequency Questionnaire was administered at Basal. The food diary was administered after the initial interview and at the end of the supplementation period. For nutritional analysis, the AvaNutri^TM^ software (Version 1.0, Rio de Janeiro, Brazil) was used based on Brazilian nutritional composition tables as a database for calculating nutrients.

**Statistical analysis:** The Shapiro–Wilk test was used to determine the normality of the data. Data were expressed as mean ± standard deviation, and sample characteristic variables were compared using one-way ANOVA. To compare group means, sleep quality, and daytime sleepiness, a two-way ANOVA with repeated measures was used, followed by Tukey’s post hoc test. Data were analyzed using SPSS Statistics 13.3, with a significance level set at *p* ≤ 0.05.

## 3. Results

During the study, one participant in the Probiotic group withdrew due to an increase in self-reported gastrointestinal symptoms after using the supplement. On the day of the marathon, one participant in the Placebo group did not complete the marathon due to personal issues, and another participant in the Placebo group dropped out at 27 km. Thus, 27 subjects completed all stages of the study (Probiotic group, n = 14; Placebo group, n = 13).

Table 1 presents comparisons of the means for the characteristics of the Probiotic and Placebo groups. There were no differences in age, body mass, or height. Table 1 also shows that there was no difference in habitual food intake between the groups at Basal and before the marathon. There was also no difference in food intake during the marathon.

Regarding the self-reported presence of gastrointestinal symptoms, 61.9% of participants reported experiencing some symptoms at some point, with 38.1% belonging to the Placebo group and 23.8% to the Probiotic group (Table 2). The most frequently reported symptoms were flatulence (Placebo group = 60%; Probiotic group = 18%), urge to defecate (Placebo group = 40%; Probiotic group = 45%) and belching (Placebo group = 30%; Probiotic group = 27%). There was no difference between the groups (Table 2).

**Figure 2:**
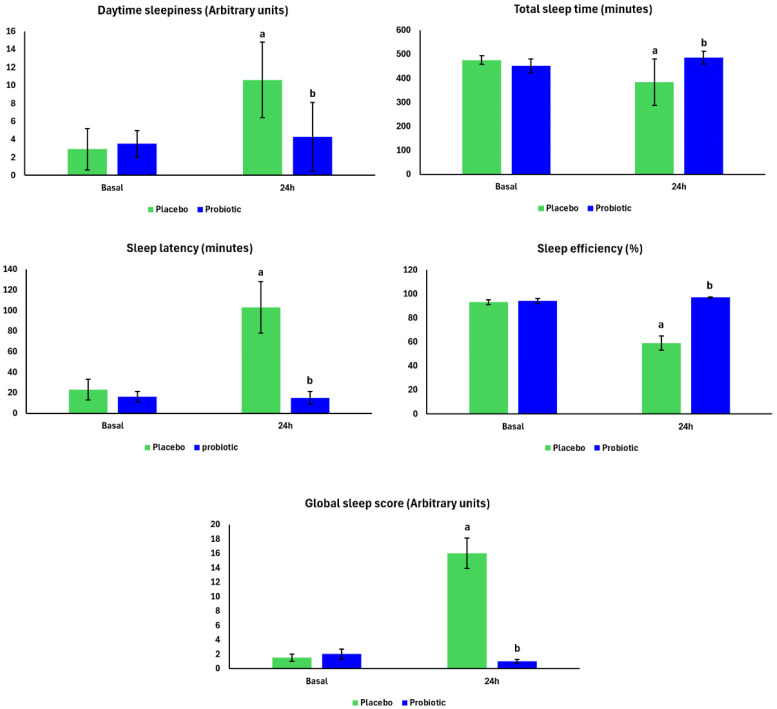
Self-reported sleep parameters in the Placebo group (n = 13) and Probiotic group (n = 14) at Basal and 24 h after the marathon. Two-way ANOVA with repeated measures and Tukey’s post hoc test. *p* < 0.05. Placebo group, green bars; Probiotic group, blue bars. “a” = different from Basal, and “b” = different from Placebo group.

Figure 2 shows the self-reported sleep parameters that were assessed. There was an increase in self-reported sleepiness 24 h after the marathon in the Placebo group compared to Basal (*p* = 0.001). In the Probiotic group, self-reported sleepiness was lower than in the Placebo group 24 h after the marathon (*p* = 0.001). Self-reported sleep latency was longer in the Placebo group 24 h after the marathon compared to Basal (*p* = 0.001). On the other hand, self-reported sleep latency was shorter in the Probiotic group compared to the Placebo group 24 h after the marathon (*p* = 0.001).

The Placebo group experienced a decrease in self-reported total sleep time (*p* = 0.05) and sleep efficiency (*p* = 0.05) 24 h after the marathon, compared to their own Basal values. No differences were found between the Basal and 24 h post-marathon measurements in the Probiotic group; however, self-reported total sleep time (*p* = 0.05) and sleep efficiency (*p* = 0.04) were higher in the Probiotic group compared to the Placebo group 24 h after the marathon. The Global Sleep Score (an index reflecting the self-reported overall quality of sleep, obtained from the weighted sum of the PSQI domains, where higher values indicate worse self-reported quality of sleep) was higher 24 h after the marathon in the Placebo group compared to the Basal group (*p* = 0.001). However, the score was lower in the Probiotic group when compared to the Placebo group 24 h after the marathon (*p* = 0.001). No differences were observed between the groups at Basal.

**Figure 3:**
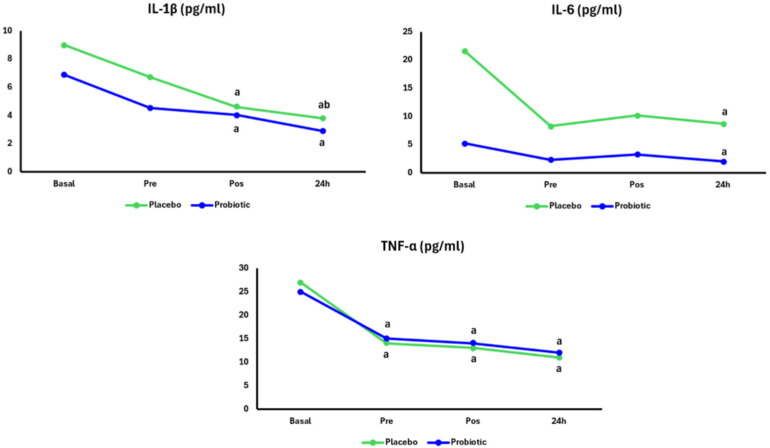
Cytokines in the Placebo group (n = 13) and Probiotic group (n = 14) at Basal and 24 h after the marathon. Two-way ANOVA with repeated measures and Tukey’s post hoc test. *p* < 0.05. Placebo group, green lines; Probiotic group, blue lines. “a” = different from the Basal moment, and “b” = different from pre.

Figure 3 shows the plasma concentration of cytokines. In the Placebo group, IL-1β levels decreased at both pos (*p* = 0.001) and 24 h (*p* = 0.001) time points compared to Basal, with a further decrease observed at 24 h compared to pre (*p* = 0.03). In the Probiotic group, a decrease was observed at the pos (*p* = 0.01) and 24 h (*p* = 0.001) time points compared to Basal. The concentration of IL-1β was not different between the groups. Regarding IL-6, in the Placebo group (*p* = 0.03) and Probiotic group (*p* = 0.04), there was a decrease at 24 h compared to Basal. There was no difference in IL-6 concentration between the groups. Regarding TNF-α, in the Placebo group, there was a decrease pre (*p* = 0.01), pos (*p* = 0.001), and 24 h after the marathon (*p* = 0.01) compared to Basal. In the Probiotic group, there were decreases in the pre (*p* = 0.01), pos (*p* = 0.001), and 24 h (*p* = 0.001) periods compared to Basal. There was no difference in TNF-α concentration between the groups.

**Figure 4:**
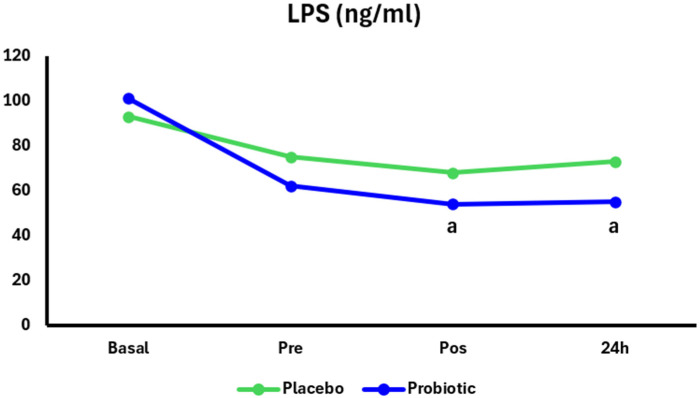
LPS in the Placebo group (n = 13) and Probiotic group (n = 14) at Basal and 24 h after the marathon. Two-way ANOVA with repeated measures and Tukey’s post hoc test. *p* < 0.05. Placebo group, green lines; Probiotic group, blue lines. “a” = different from the Basal moment.

Regarding LPS concentration, in the Probiotic group, there was a significant decrease in the pos (*p* = 0.01) and 24 h (*p* = 0.01) periods compared to Basal. No significant differences were found between the groups in LPS concentration (Figure 4).

## 4. Discussion

The main finding of the study was that supplementation with *Lactobacillus acidophilus* and *Bifidobacterium lactis* for 30 days appeared to preserve self-reported sleep quality 24 h after the marathon, relative to the placebo, within the limits of subjective assessment. This effect was accompanied by lower serum concentrations of lipopolysaccharides (LPSs), suggesting preservation of intestinal integrity and modulation of the inflammatory response. This is one of the first studies to evaluate the effects of strenuous exercise and probiotic supplementation on self-reported sleep. The results suggest that probiotic supplementation may influence self-reported sleep after strenuous exercise, such as a marathon.

No significant differences were observed between the Placebo and Probiotic groups in terms of self-reported habitual food intake or food intake during the marathon. Therefore, the groups presented equivalent dietary patterns, both in terms of macronutrient composition and energy intake, which reinforces the homogeneity of the sample and reduces the likelihood that dietary differences influenced the observed changes in sleep and inflammation parameters.

In this study, the general macronutrient content of the diet did not differ significantly between groups, suggesting that outcomes may be attributable to the treatment rather than the background diet [24,25]. The absence of differences in this aspect supports the interpretation that the beneficial effects observed in the Probiotic-supplemented group were predominantly due to the proposed intervention rather than to prior or acute dietary differences associated with the marathon.

At Basal, no differences were found between the Probiotic and Placebo groups, suggesting that any changes foreseen were due to the supplementation and/or the physiological stress. The change in self-reported sleep in the Placebo group is consistent with previous studies on the impact of prolonged exercise on sleep. Strenuous physical exertion, such as marathon running, activates the hypothalamic–pituitary–adrenal axis and increases cortisol release, which can delay sleep onset and reduce total sleep time [3,4,26]. These effects of exercise on sleep, combined with increased body temperature and sympathetic activation, worsen sleep and contribute to the daytime sleepiness observed after marathon running [13,17]. These effects characterize a transient neuroendocrine response sufficient to compromise circadian balance and physiological recovery.

The self-reported sleep parameters of runners supplemented with probiotics on the first night after the marathon differed from those of the Placebo group, indicating that 30 days of supplementation with *Lactobacillus acidophilus* and *Bifidobacterium lactis* may create a protective environment, resulting in changes to self-reported sleep and preserved barrier function in the gastrointestinal tract. The reduction in LPS observed only in the Probiotic group is particularly relevant, given that this membrane component of Gram-negative bacteria is a significant trigger of systemic inflammation associated with intense exercise [9,10].

During endurance exercise, the redistribution of blood flow to active muscles and the increase in core temperature compromise splanchnic perfusion, leading to intestinal barrier dysfunction and the passage of LPS into the circulation [27,28]. LPS, when binding to Toll-like receptor 4 (TLR4) on immune and endothelial cells, induces the release of pro-inflammatory cytokines, amplifying the systemic inflammatory response [29,30].

Increased intestinal permeability and elevated LPS levels after exercise are associated with poorer sleep [31]. The present study indicates that supplementation with *Lactobacillus acidophilus* and *Bifidobacterium lactis* may attenuate this process. Treatment with both species has been described as strengthening epithelial junctions, increasing mucin production, and reducing the adhesion of pathogenic bacteria [19,32]. Furthermore, the modification of the intestinal microbiota resulting from the supplementation period may lead to increased production of short-chain fatty acids, such as butyrate, which has a notable anti-inflammatory effect and contributes to maintaining intestinal barrier integrity [5,33]. Thus, the lower LPS concentration observed in supplemented athletes may reflect a more intact gut and a more controlled inflammatory response.

In the present study, greater control of the inflammatory profile may lead to changed self-reported sleep on the first night after the marathon. However, self-reported sleep changes may be much more closely associated with reduced LPS levels than with changes in other inflammatory mediators investigated in the Probiotic group. Elevated IL-1β and TNF-α concentrations alter the expression of genes involved in regulating the circadian rhythm and the metabolism of sleep-related neurotransmitters, such as serotonin and GABA [34,35]. The reduction in these mediators, as observed compared to Basal, combined with reduced LPS translocation, may have created a favorable environment for preserving the sleep parameters analyzed in supplemented athletes. This integrative “protective sleep” effect suggests a three-pronged support system for changes in self-reported sleep patterns the night after the marathon, comprising a better balance of pro- and anti-inflammatory factors, immunometabolism, and neurochemical factors.

Changes in IL-1β, IL-6, and TNF-α concentrations require cautious interpretation. Previous studies have shown that the most significant increase in IL-6 occurs during exercise, with the peak typically occurring at the end of the exercise. However, the increase in TNF-α and IL-1β is more discreet and delayed than for IL-6. Several factors may contribute to the increase in cytokines during exercise, including exercise intensity and volume, the muscle mass involved in the exercise, and the individual’s training level [11,36]. Nutritional status also significantly influences cytokine production [37]. However, the lack of differences between the two groups regarding body composition, dietary pattern, and food consumption during the marathon may explain the changes in cytokines at different times, but not between groups.

In the present study, a reduction in IL-1β, IL-6, and TNF-α was observed compared to the Basal levels, but there were no differences between the groups. The pattern of results found may reflect the timing of collections (Basal, pre, pos, and 24 h), with no change immediately after the marathon. Alternatively, the reduction in all three cytokines may indicate a rebound anti-inflammatory effect or an after-marathon regulatory response, especially in well-trained athletes whose acute inflammatory response tends to be attenuated [38].

The circadian rhythmicity of pro-inflammatory cytokines may have influenced the pattern of results observed in the present study. Several cytokines exhibit variation in circulation throughout the day, notably IL-6, as some studies have shown peaks in the early morning hours (around 2–6 a.m.). In contrast, others detect peaks in the late afternoon or evening, while the concentration of TNF-α and IL-1β also exhibits circadian modulation in humans [39]. Considering that the Basal, pre, and 24 h samples in this study were collected after an overnight fast between 7:00 and 8:00 a.m. In contrast, the “pos” sample was collected between 10:00 and 11:00 a.m. (i.e., at varying times depending on individual arrival time); comparisons that include the point immediately after the marathon may be affected by both the acute response to exercise and the underlying circadian fluctuations in cytokine concentrations. Taken together, the reductions in IL-1β, IL-6, and TNF-α compared to basal levels reported here may reflect a combination of post-exercise regulatory dynamics and the normal circadian decline, rather than just a possible anti-inflammatory effect of running [40].

The possible protective effect of probiotics observed in this study confirms previous results linking microbiota modulation to changes in sleep [17,19]. However, it is more dependent on LPS concentrations than on cytokines. Although the literature on athletes is limited, there is evidence that regular probiotic consumption reduces inflammatory markers and symptoms of fatigue after endurance events [41,42]. Our findings expand this knowledge by showing that the benefit may also extend to sleep regulation on the first night after the marathon. This variable has not been extensively studied but is crucial for recovery and performance in exercisers [43].

From a practical perspective, the results suggest that supplementation with *Lactobacillus acidophilus* and *Bifidobacterium lactis* can serve as a non-pharmacological nutritional strategy to preserve self-reported sleep and alter the inflammatory profile after endurance competitions, thereby expanding the potential effects of these two probiotics. This approach is low-cost, safe, and potentially valuable for preventing overtraining in programs or contexts of high physical demand, such as military missions and prolonged training and endurance competitions. The change in sleep quality on the first night after the marathon can, in turn, have a positive effect on immunity, energy metabolism, and subsequent performance [44,45].

Some limitations should be considered. Participants were restricted to men, which limits the generalizability of the results. Despite the applicability of questionnaires, sleep was not assessed by objective measures such as actigraphy or polysomnography. Details are lacking regarding nutritional intake, such as micronutrients, polyphenols, and sources of macronutrients. Furthermore, the absence of fecal sample analysis would prevent the direct identification of changes in microbial composition. Future studies should include integrated omics analyses (microbiome, metabolome, and transcriptome) to more precisely elucidate the mechanisms involved in the interaction between microbiota, inflammation, and sleep regulation after intense exercise. Further studies should also be conducted to assess whether other combinations of probiotics or different supplementation times can confirm or contradict the results found in this study.

## 5. Conclusions

The marathon changed the self-reported sleep parameters investigated. In contrast, supplementation with *Lactobacillus acidophilus* and *Bifidobacterium lactis* for 30 days attenuated these self-reported effects on the first night after the marathon, possibly by reducing LPS and limiting the activation of inflammatory pathways. These findings suggest the role of probiotics in post-exercise recovery, indicating that modulating the gut microbiota may be an effective tool for protecting sleep and mitigating the inflammatory impact of prolonged exercise in endurance athletes.

## Figures and Tables

**Table 1:** Descriptive data of the sample, food intake during the marathon, and usual food intake.

	**Descriptive Data of the Sample**			
	Placebo (n = 13)		Probiotic (n = 14)	
Age (Years)	40.46 ± 7.79		35.96 ± 5.81	
Stature (cm)	1.75 ± 0.08		1.75 ± 0.06	
Body Mass (kg)	74.12 ± 10.20		77.36 ± 10.99	
	**Self-reported food consumption during the marathon**			
	Placebo (n = 13)		Probiotic (n = 14)	
Energy (Kcal)	526.36 ± 317.97		678.20 ± 172.88	
Carbohydrates (g)	109.07 ± 60.74		137.66 ± 112.66	
Proteins (g)	4.60 ± 8.47		9.49 ± 29.55	
Fat total (g)	7.98 ± 16.52		11.41± 28.33	
Fat saturated (g)	0.79 ± 2.57		2.46 ± 8.44	
Fat Monounsaturated (g)	0.12 ± 0.38		0.94 ± 3.47	
Fat polyunsaturated (g)	0.23 ± 0.69		0.44 ± 1.54	
Cholesterol (mg)	0		24.98 ± 93.29	
Fibers (g)	1.58 ± 1.76		2.30 ± 4.20	
	**Self-reported usual food consumption**			
	Basal		Pre-marathon	
	Placebo (n = 13)	Probiotic (n = 14)	Placebo (n = 13)	Probiotic (n = 14)
Energy (Kcal)	2514.9 ± 962.95	2514.91 ± 962.95	2282.58 ± 577.50	2282.58 ± 577.50
Carbohydrates (g)	342.78 ± 162.03	385.23 ± 197.14	317.32 ± 142.94	342.86 ± 164.30
Proteins (g)	106.90 ± 44.46	136.50 ± 97.96	88.85 ± 21.08	128.17 ± 30.70
Fat total (g)	104.0 ± 48.50	94.60 ± 40.39	74.64 ± 38.28	81.49 ± 34.61
Fat saturated (g)	36.81± 16.69	29.52 ± 22.40	24.24 ± 19.42	23.71± 10.51
Fat Monounsaturated (g)	21.62 ± 14.16	21.62 ± 14.16	11.31± 5.34	14.86 ± 6.96
Fat polyunsaturated (g)	8.66 ± 6.15	8.66 ± 6.15	13.17 ± 10.67	6.67 ± 4.66
Cholesterol (mg)	474.41 ± 250.86	474.41 ± 250.86	377.64 ± 363.44	491.13 ± 397.00
Fibers (g)	23.65 ± 10.53	25.63 ± 15.61	24.92 ± 19.15	15.61 ± 15.25

Descriptive data of the sample, self-reported food intake during the marathon, and self-reported usual food intake in the Placebo group (n = 13) and Probiotic group (n = 14) at Basal and pre moments. Two-way ANOVA with repeated measures and Tukey’s post hoc test.

**Table 2:** Description of self-reported gastrointestinal symptoms during the study.

	Total		Placebo	Probiotic
Presence of symptoms	Yes (61.9%)	No (38.1%)	38.1%	23.8%
	Placebo		Probiotic	
	Sometimes	Always	Sometimes	Always
Stomach problems	10%	-	27%	-
Nausea	0%	-	0%	-
Dizziness	10%	-	9%	-
Flatulence	60%	-	18%	9%
Urge to defecate	40%	-	45%	-
Burping	30%	-	27%	-
Heartburn	0%	-	9%	-
Bloating	10%	-	18%	-
Pain Stomach	10%	-	18%	-
Pain Intestinal	0%	-	9%	-
Feeling of wanting to vomit	20%	-	18%	-
Vomiting	0%	-	18%	-
Diarrhea	0%	-	0%	-

## Data Availability

The data presented in this study are available on request from the corresponding author due to privacy.
